# Supplementary material for: The PD-L1 Expression and Tumor-Infiltrating Immune Cells Predict an Unfavorable Prognosis in Pancreatic Ductal Adenocarcinoma and Adenosquamous Carcinoma
Source: J Clin Med. 2023 Feb 9;12(4):1398. doi: 10.3390/jcm12041398 (PMC9965576; doi:10.3390/jcm12041398)
Supplement: Supplementary file 1 [file jcm-12-01398-s001.zip › jcm-2157896-supplementary.pdf]

Supplementary Materials

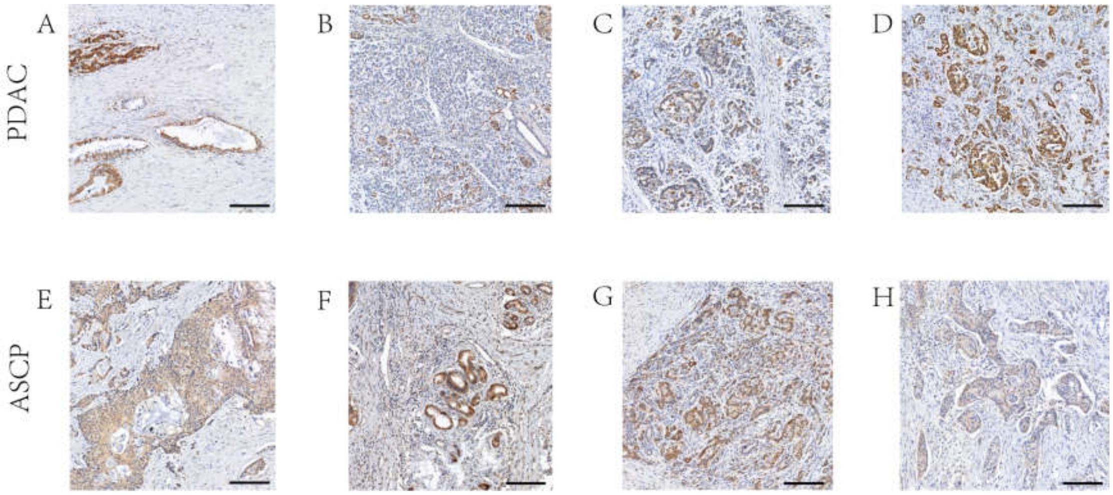

Figure S1: Representative pictures for PD-L1 immunostaining

Representative pictures for PD-L1 immunostaining (brown) in PDAC (A-D) and ASPC (E-H). Magnification, 40×; scale bars, 50 μm.

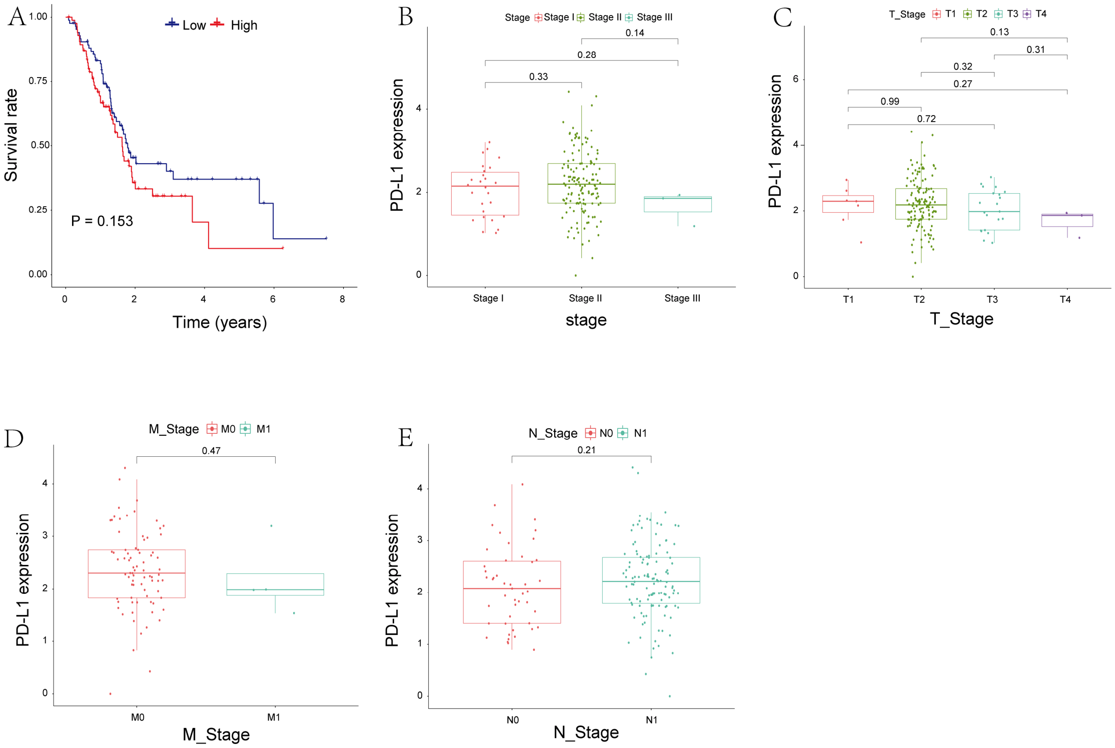

Figure S2: The expression of PD-L1 correlation with survival and clinicopathological staging characteristics of PDAC patients from TCGA datasets

(A) Survival analysis for PDAC patients with different PD-L1 expression. Patients were marked with high expression or low expression depending on comparing with the median expression level. p = 0.153 by log-rank test. (B–E) The correlation of PD-L1 expression with clinicopathological characteristics. Wilcoxon rank sum or Kruskal–Wallis rank sum test acted as the statistical significance test.

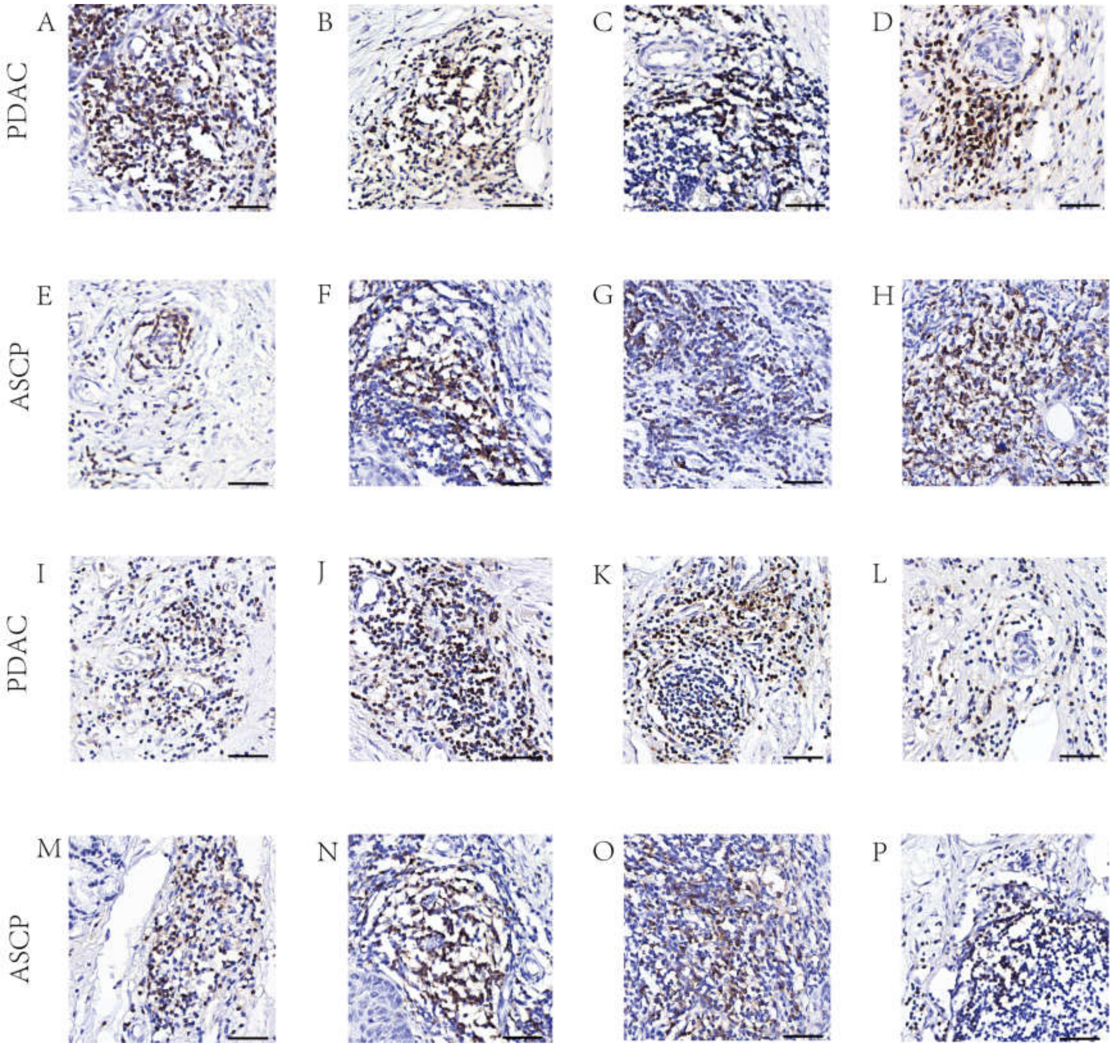

Figure S3: Representative pictures for CD3 and CD4 immunostaining
(A-H) Representative pictures for CD3 immunostaining (brown) in PDAC (A-D) and ASPC (E-H). Magnification, 40×; scale bars, 50 μm. (I-P) Representative pictures for CD4 immunostaining (brown) in PDAC (I-L) and ASPC (M-P). Magnification, 40×; scale bars, 50 μm.

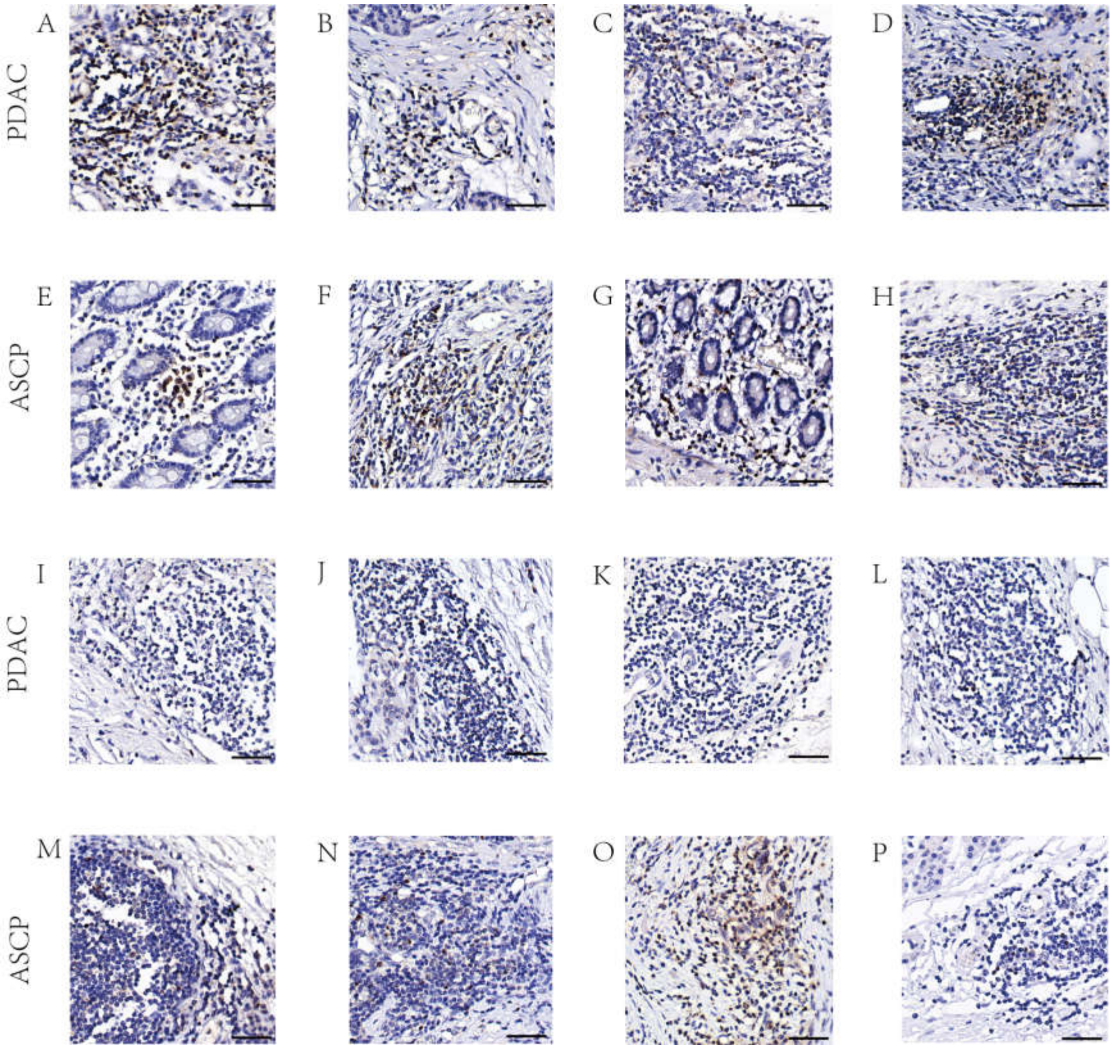

Figure S4: Representative pictures for CD8 and FoxP3 immunostaining
(A-H) Representative pictures for CD8 immunostaining (brown) in PDAC (A-D) and ASPC (E-H). Magnification, 40×; scale bars, 50 μm. (I-P) Representative pictures for FoxP3 immunostaining (brown) in PDAC (I-L) and ASPC (M-P). Magnification, 40×; scale bars, 50 μm.

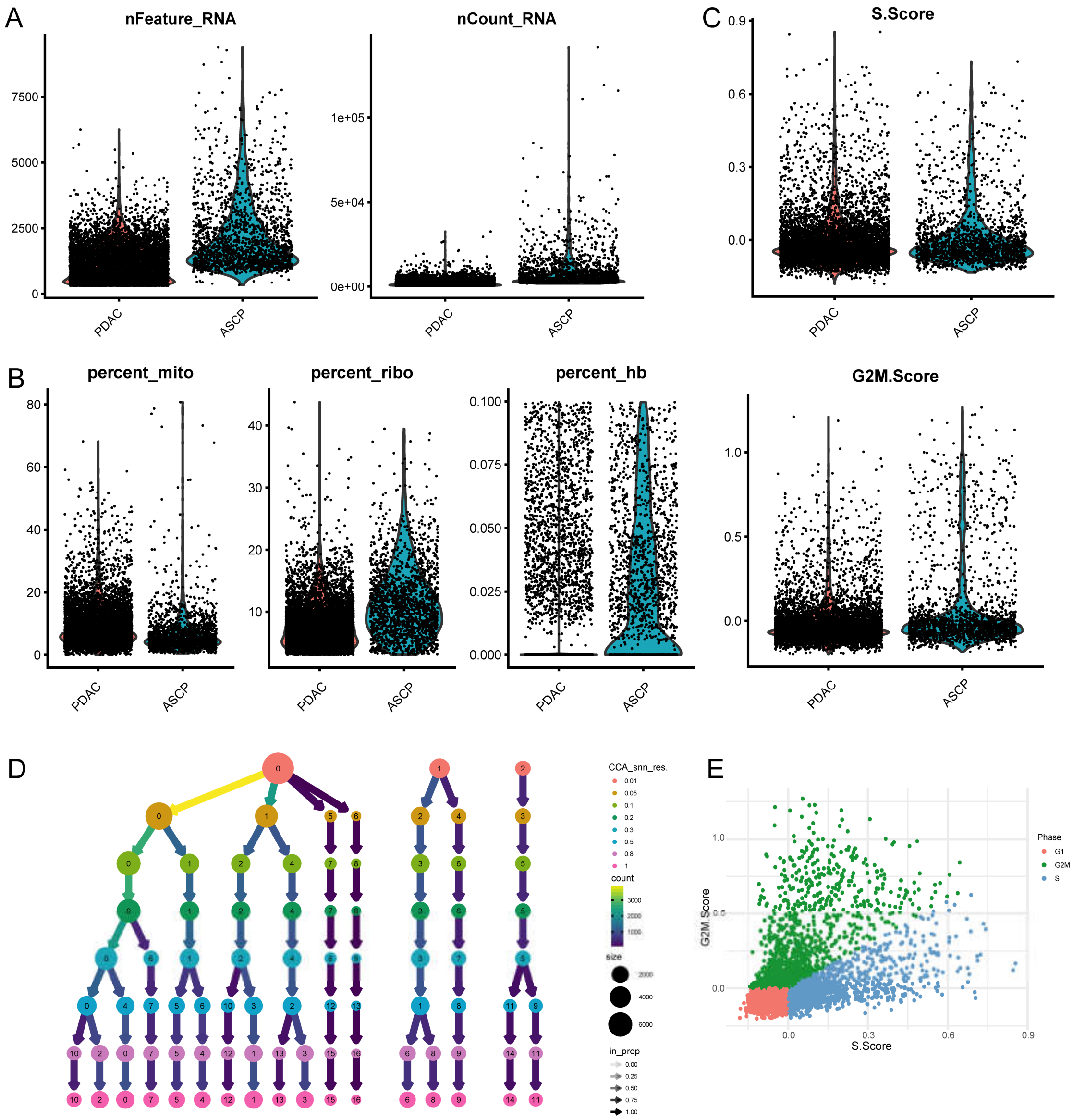

Figure S5: Quality control result and cell cycle score

(A) The percent of detected genes from single-cell data of PDAC and ASCP. Samples with fewer than 500 genes, more than 5,000 genes, and fewer than 3 cells/gene were removed. (B) The percent of mitochondrial, ribosomal, and hemoglobin genes. Samples with fewer than 20% mitochondrial genes, fewer than 20% ribosomal genes, and fewer than 10% hemoglobin genes were selected. (C) The cluster tree of subgroup amounts shows different resolution ratios, and 0.8 was chosen for subsequent analysis. (D) Cluster tree of subgroup amounts at different resolution ratios. (E) The number of G1, G2M, and S phase cells. Remove cell cycle effects according to the cell cycle phase scores.

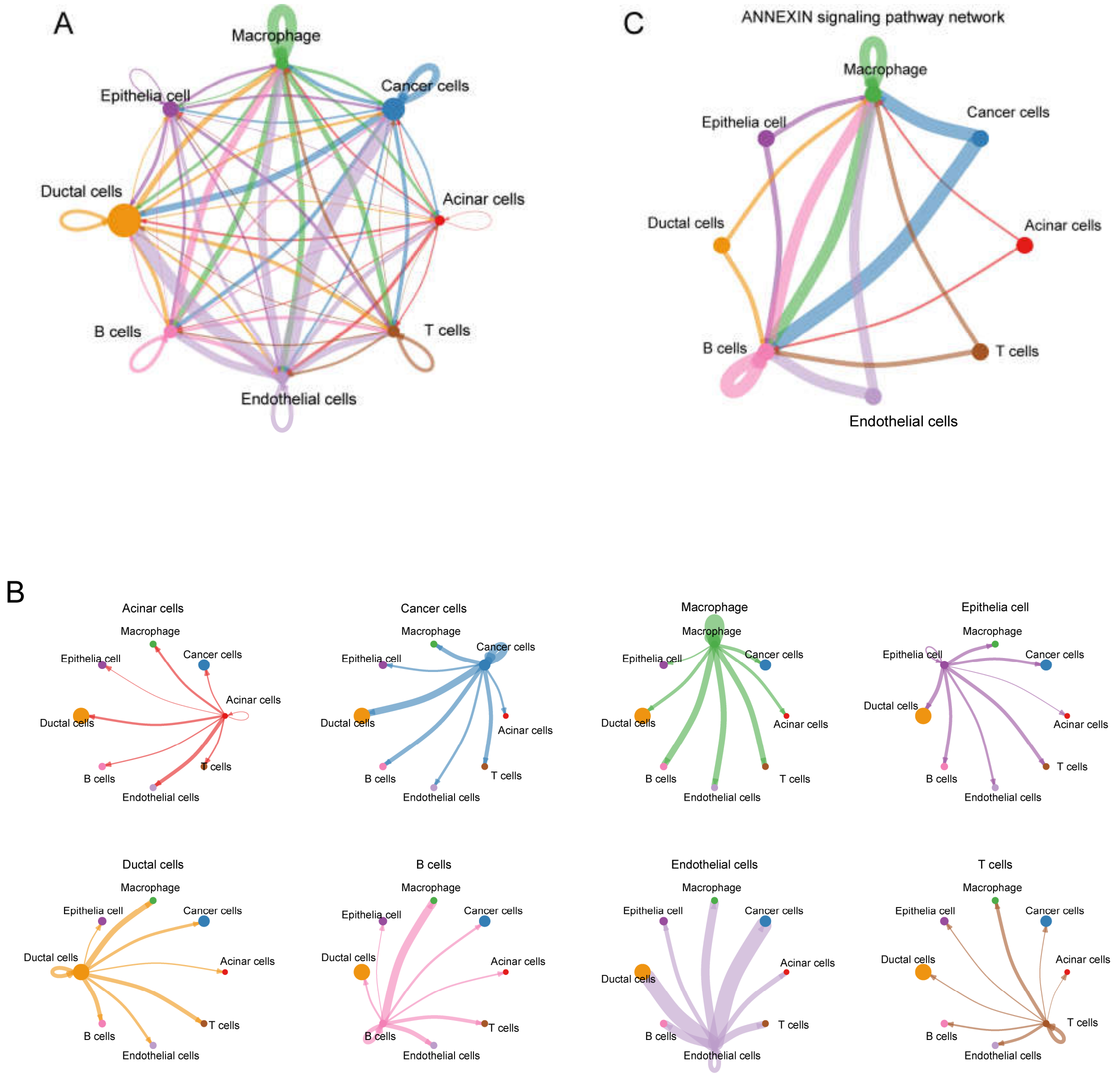

Figure S6: The CellChat between each subgroup.

The multiple pathways in tumor cells, such as the ANNEXIN signaling pathway, could represent new targets for immunotherapy. (A-B) CellChat has been used to quantitively infer and analyze intercellular communication networks from scRNA-seq data. (C) The ANNEXIN signaling pathway network in each subgroup.

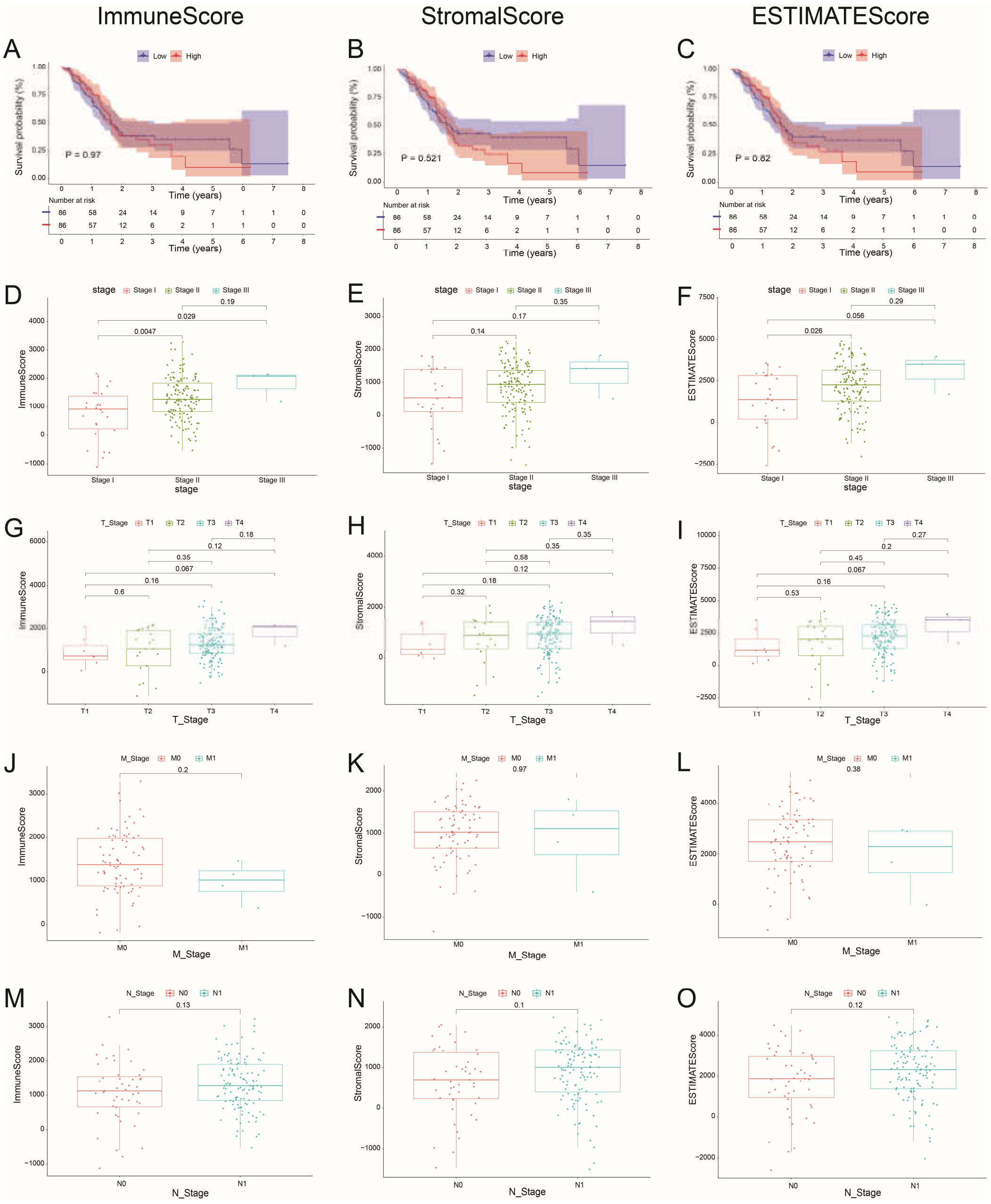

Figure S7: Correlation analyses of scores with the survival and clinicopathological characteristics of PDAC patients

(A–C) Kaplan–Meier survival analysis for PDAC patients grouped into high or low scores in ImmuneScore, StromalScore, and ESTIMATEScore determined by comparing them with the median. p = 0.97, 0.52, and 0.82, respectively, by log-rank test. (D–F) Distribution of ImmuneScore, StromalScore, and ESTIMATEScore in the stage classification. (G–I) Distribution of three kinds of scores in the T classification by Kruskal–Wallis rank sum test for ImmuneScore, StromalScore, and ESTIMATEScore, respectively. (J–L) Distribution of scores in the M classification (p = 0.20, 0.97, 0.38 by Wilcoxon rank sum test for ImmuneScore, StromalScore, and ESTIMATEScore separately). (M–O) Distribution of scores in N classification. Similar to the preceding, p = 0.13, 0.10, 0.12, respectively, with Wilcoxon rank sum test.

Table S1. Clinicopathological characteristics statistics of PDAC patients from TCGA datasets.

Clinical characteristics		TCGA datasets (n = 169)	
		n	%
Age(years)	⩾65	93	55.03
	<65	76	44.97
Stage	I	18	10.65
	II	143	84.62
	III	3	1.78
	IV	5	2.96
T Stage	T1	6	3.55
	T2	21	12.40
	T3	139	82.20
	T4	3	1.78
N Stage	N0	47	27.81
	N1	119	70.41
	NX	3	1.78
M Stage	M0	78	46.15
	M1	5	2.96
	MX	86	50.89
OS Times (Months)	⩾12	112	66.27
	<12	57	33.73

Table S2. The cutoff values (cells/mm2) of CD3+, CD4+, CD8+, and FoxP3+ cells

Cutoff values (cells/mm2)	ASCP	PADC	PC
CD3+	312	177	194
CD4+	239	134	134
CD8+	148	197	197
FoxP3+	27	33	33

Table S3. The number and percentage of patients in each group

	ASCP(n=29)		PDAC(n=54)		PC(n=83)	
	Low expression	High expression	Low expression	High expression	Low expression	High expression
PD-L1	19(65.52)	10 (34.48)	37(68.52)	17(31.48)	53(63.86)	30(36.14)
CD3	12(41.38)	17(58.62)	13(24.07)	41(75.93)	24(28.92)	59(71.08)
CD4	12(41.38)	17(58.62)	23(42.59)	31(57.41)	27(32.53)	56(67.47)
CD8	19(65.52)	10(34.48)	25(46.30)	29(53.70)	47(56.63)	36(43.37)
FoxP3	9(31.03)	20(68.97)	33(61.11)	21(38.89)	43(51.81)	40(48.19)

The percentage of patients are shown within the parenthesis.
